# Bovine blood products modulate the effect of ophthalmic antibiotics against common bacterial pathogens in dogs with infectious keratitis

**DOI:** 10.3389/fvets.2026.1880844

**Published:** 2026-09-28

**Authors:** Helen O. Kim, Melissa A. Kubai, Levi Brock, Chloe C. Stinman, Janelle Gapp, Danyang Zhang, Rachel A. Allbaugh

**Affiliations:** 1 Department of Veterinary Clinical Sciences, Iowa State University, College of Veterinary Medicine, Ames, IA, United States; 2 Veterinary Diagnostic Laboratory, Iowa State University, College of Veterinary Medicine, Ames, IA, United States; 3 Department of Veterinary Diagnostics and Production Animal Medicine (Statistics), Iowa State University, College of Veterinary Medicine, Ames, IA, United States

**Keywords:** alpha-2 macroglobulin, antibiotic resistance, antimicrobial susceptibility testing, bacterial keratitis, blood derivatives, minimum inhibitory concentrations, protein binding

## Abstract

Bacterial keratitis is a common condition that can result in serious complications including vision impairment and loss of the eye if not treated aggressively. Topical blood products are often utilized concurrently with topical antibiotics for their known anti-collagenolytic properties. These blood products contain albumin that could affect the antibiotic efficacy due to drug-protein binding. In this study, serum and plasma were harvested from 10 healthy bovines, utilizing fresh and frozen (1 month at −20 °C) aliquots for the experiment. Albumin and alpha-2 macroglobulin levels were quantified using species-specific ELISA kits. 30 bacterial isolates (10 *Staphylococcus pseudintermedius*, 10 *Streptococcus canis*, 10 *Pseudomonas aeruginosa*), isolated from canine patients with infectious keratitis were tested with blank plates as well as commercial susceptibility plates (Sensititre™ JOEYE2) to assess the minimal inhibitory concentration (MIC) of 17 different antibiotics in the absence (control) or presence of serum or plasma (fresh or frozen) from bovines (4 test groups). Mean bovine albumin levels ranged from 1.98 to 2.00 mg/ml. The median bovine alpha-2 macroglobulin levels were similar between frozen serum (4,403 ng/ml [range 3,542–5,499 ng/ml]) and frozen plasma (4,959 ng/ml [range 3,305–5,447 ng/ml]). No bacteriological susceptibility differences were noted between fresh and frozen samples for either serum or plasma. Bovine serum/plasma had direct antimicrobial effects in selected bacterial isolates (up to 49% with frozen plasma for *Staphylococcus pseudintermedius*). When compared with control, bovine serum/plasma increased MICs (1.33–4-fold) or decreased MICs (0.5–0.75 fold) depending on the specific antibiotic and bacterial isolate. In practice, clinicians should consider using bovine serum/plasma over other species' blood products (equine or canine) given direct antimicrobial effects, good anti-collagenolytic potential, and lesser impact on antimicrobial susceptibility to topical antibiotics. Serum and plasma can alter antibiotic efficacy (antibiotic-protein binding from high levels of albumin); therefore, serum/plasma should be applied last and spaced ≥15 min after topical antibiotics.

## 1 Introduction

Bacterial keratitis is a global cause of ocular discomfort leading to potential vision loss in many species including dogs, cats, horses and humans (1–4). Following corneal injury (e.g., secondary to trauma or exposure), ulcers can develop a secondary infection due to bacterial introduction or the presence of local microflora on the ocular surface which can turn pathogenic in compromised eyes. Despite appropriate aggressive medical therapy, these eyes can develop negative sequelae such as keratomalacia (“corneal melting”), fibrosis, corneal perforation, loss of vision, and loss of the eye.

Medical management for bacterial keratitis requires many topical therapeutics, which may include antimicrobial therapy, atropine, and blood products (serum or plasma). Blood products are used to promote corneal wound healing as they contain numerous growth factors (1). Serum and plasma are potent anti-collagenolytic agents (prevent corneal melting) by inhibiting endogenous proteinases and matrix metalloproteinases (MMPs) released by bacteria, neutrophils and keratocytes (5, 6). However, these blood products contain high levels of albumin (7), which reduces topical antimicrobial efficacy by binding to free antibiotics, rendering only the unbound portion of the antibiotic microbiologically active (8, 9). Sebbag et al. showed that serum albumin increased the minimum inhibitory concentrations (MICs) of 17 topical antibiotics in a bacteria-specific, dose-dependent and antibiotic-specific manner (10). More recently, Kubai et al. demonstrated that canine and equine serum and plasma (fresh vs. frozen) had high albumin concentrations (canine = 14 mg/ml, equine = 26 mg/ml), and showed a bacteria-specific and antibiotic-specific response (11). It was noted that when grouping the results for all blood products and bacterial isolates, the median fold changes in MICs were significantly greater with canine blood products compared to equine blood products. Interestingly, there was a direct antimicrobial efficacy (up to 97%) noted in the equine serum control plates when plated with *Staphylococcus pseudintermedius* (11).

The main objective of this study was to evaluate the effects of bovine blood products (plasma and serum) on the efficacy of topical antibiotics against common canine ocular pathogens (*Staphylococcus pseudintermedius, Streptococcus canis, Pseudomonas aeruginosa*) (12). A secondary objective was to determine the direct antimicrobial effect of bovine blood products, albumin concentration, and alpha-2 macroglobulin concentration (anti-melting agent) in bovine blood products in two storage conditions (e.g., frozen and fresh) to mimic use of blood products in general veterinary ophthalmology practice.

## 2 Materials and methods

### 2.1 Serum and plasma

#### 2.1.1 Collection

The study was reviewed and approved by the Institutional Animal Care and Use Committee at Iowa State University (protocol # 23-005). Samples were obtained from 10 healthy Holstein cows with informed consent from a privately owned herd in Iowa. Animals were excluded if a topical or systemic antibiotic was administered in the last 30 days. Each animal underwent two sessions of blood collection separated by 1 month, freezing the blood products at −20 °C following for the first session (frozen samples) and refrigerating the blood products at 4 °C for < 24 h following the second session (fresh samples) until the *in vitro* experiments were performed. Briefly, 30 ml of blood was collected via venipuncture using the tail vein from each animal at each session, splitting the collected blood into plain red-top tubes or heparinized green-top tubes to obtain serum or plasma following tube centrifugation, respectively.

#### 2.1.2 Albumin quantification

Albumin levels were quantified in each sample (*i.e.*, bovine fresh or frozen; serum or plasma) and run in duplicate using species-specific ELISA kits from Bethyl Laboratories Inc. (Bovine E11-113-230130) according to the manufacturer's instructions. Quality control was performed and numerous dilutions (1:100, 1:10,000, 1:250,000, 1:500,000, 1:1 million, 1:2 million) were performed to determine the appropriate dilution for bovine albumin of 1:250,000 for sample processing.

#### 2.1.3 Alpha-2 macroglobulin quantification

Alpha-2 macroglobulin levels were quantified in each sample (bovine frozen serum or plasma) and run in duplicate using a species-specific ELISA kit from Novus Biologicals Inc. (Bovine NBP2-77018) according to the manufacturer's instructions. Quality control was performed to determine the resulting dilution of 1:500 for sample processing.

### 2.2 Antimicrobial susceptibility testing

30 bacterial isolates were tested, representative of the three most common bacterial species identified in canine patients with ulcerative keratitis, specifically *Staphylococcus pseudintermedius* (*n* = 10), *Streptococcus canis* (*n* = 10), and *Pseudomonas aeruginosa* (*n* = 10).12 Bacterial isolates cultured from clinical canine patients were revived from the −80 °C ± 2 °C freezer by thawing at room temperature and grown on tryptic soy agar with 5% sheep blood agar plates. All plates were incubated at 35 ± 2 °C with 5%−10% CO2 for a total time of 24–48 h. Each bacterial isolate was then diluted to 1.5 x 10^8^ colony forming units per milliliter (CFU/mL) in sterile physiological saline using a nephelometer. Purity of the isolates were confirmed by plating diluted bacterial isolates onto blood agar plates.

Four blood products were tested: bovine serum fresh (bS-Fsh), bovine plasma fresh (bP-Fsh), bovine serum frozen (bS-Frz), bovine plasma frozen (bP-Frz). Two sets of experiments were performed (Figures 1A, B):

(1) Control plate: One blank plate (Corning 96-well Clear Polystyrene Microplates, Corning Inc.) with no antibiotics was used for each bacterial species. Bacterial broth was manually pipetted into all wells along with 25-μl of blood product in duplicates for columns 1–10 (Bovine: bS-Fsh, bP-Fsh, bS-Frz, bP-Frz) and 25-μl of sterile saline for columns 11 and 12 (positive control) (Figure 1A).
(2) Antimicrobial susceptibility plates: Each bacterial broth was transferred into glass tubes containing an equal volume of sterile saline (control) or blood product (bS-Fsh, bP-Fsh, bS-Frz, bP-Frz). Then, an automated inoculation delivery system (Sensititre AIM^TM^, Thermo Scientific Inc.) was used to transfer each broth/blood product solution onto standard plates (50-μl in each of the 48 wells) that are specifically manufactured for sensitivity testing in ophthalmology (Sensititre^TM^ JOEYE2 plate, Thermo Scientific Inc.). These plates are preloaded with serial dilutions of 17 different antibiotics, which included the following common topical antibiotics: bacitracin, chloramphenicol, cefazolin, erythromycin, gentamicin, neomycin, ofloxacin, oxytetracycline, and tobramycin (assets.thermofisher.com/TFS-Assets/MBD/Specification-Sheets/Sensititre-Plate-Layout-JOEYE2.pdf). A total of 3 JOEYE2 plates were used for each bacterial isolate, which allowed to test in duplicates (Figure 1B). Following CLSI guidelines, all positive control plates and Sensititre^TM^ plates were incubated at 35 °C ± 2 °C for either 16–20h (*Pseudomonas sp*.), 20–24h (*Streptococcus sp*.), or 24 h (*Staphylococcus sp*.), followed by data recording using a digital MIC viewing system (Sensititre Vizion^TM^, Thermo Scientific Inc.). The presence or absence of bacterial growth was recorded in each well of the blank plates. For each of the 17 antibiotics in JOEYE plates, the MIC was read as the lowest concentration of antimicrobial agent that completely inhibited organism growth, a result that was accompanied by a clinical interpretation (*i.e.*, susceptible, intermediate, resistant, or non-interpretable) based on the breakpoints described in the VET08 and M100 CLSI documents (13, 14).

**Figure 1:**
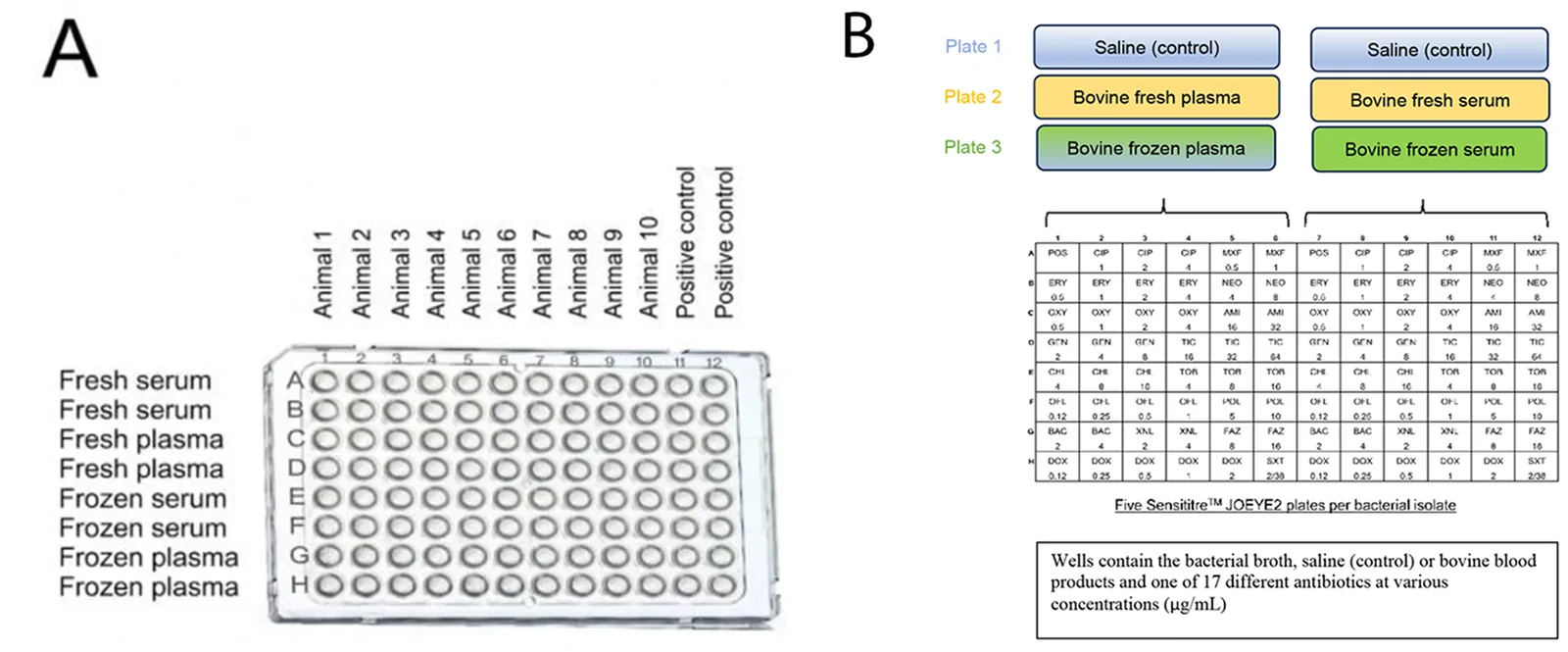
Graphical representation of the study design involving 30 bacterial isolates from canine patients with infectious keratitis (10 *Staphylococcus pseudintermedius*, 10 *Streptococcus canis*, 10 *Pseudomonas aeruginosa*) and blood products (serum or plasma, fresh or frozen) retrieved from healthy bovines. **(A)** One blank plate was used for each bacterial species, with wells containing the bacterial broth and bovine blood products, but no antibiotics; tested in duplicate. **(B)** Three Sensititre^TM^ JOEYE2 plates per bacterial species with wells containing the bacterial broth, saline (control) or bovine blood products and one of 17 different antibiotics at various concentrations (μg/ml).

### 2.3 Data analysis

A mixed-effects linear model was fitted using the lmer4 package (version 1.1–35.1) to evaluate the effect of bovine blood product type (serum vs. plasma) and storage condition (fresh vs. frozen) on albumin concentration. Bovine sample ID was included as a random effect. Blood product type, storage condition, and their interaction were included as fixed effects. Statistical significance was then assessed using Type III ANOVA conducted on the fitted model. A paired *t*-test was used to compare the concentration of A2M in frozen serum and frozen plasma samples. MIC fold change comparisons among the four blood product types (bP-Fsh, bS-Fsh, bP-Frz, and bS-Frz) were performed for each bacterial species and antibiotics that showed variation in MIC fold changes across blood product types. The Friedman rank sum test was used to account for within-sample dependence and non-normality. If the Friedman test was significant, a *post-hoc* Nemenyi test was conducted for pairwise comparisons, with family-wise error rate control to account for multiple comparisons. Tests were only performed for antibiotics with different MIC fold changes among blood products.

Statistical analyses were performed with R (v.4.3.3) and *P* values < 0.05 were considered significant.

## 3 Results

### 3.1 Albumin levels

Albumin levels in bovine blood products are summarized in Figure 2. Bovine plasma and serum samples contained albumin concentrations ranging from 1.93 to 2.24 mg/ml with no statistically significant difference between serum and plasma albumin levels (*P* = 0.272) nor fresh and frozen samples (*P* = 0.452).

**Figure 2:**
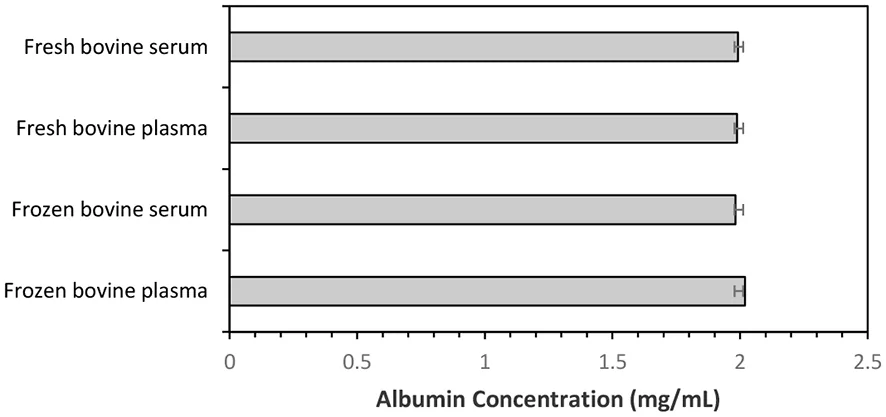
Bar chart depicting the mean ± standard deviation concentration of albumin (mg/ml) in serum and plasma of 10 healthy bovine, as quantified in fresh and frozen samples.

### 3.2 Alpha-2 macroglobulin levels

Alpha-2 macroglobulin (A2M) levels in frozen bovine blood products are summarized in Figure 3. Median alpha-2 macroglobulin concentrations were significantly higher (*P* = *0.003*) in frozen plasma (4,959 ng/ml [range 3,305–5,447 ng/ml]) compared to frozen serum (4,403 ng/ml [range 3,542–5,499 ng/ml]).

**Figure 3:**
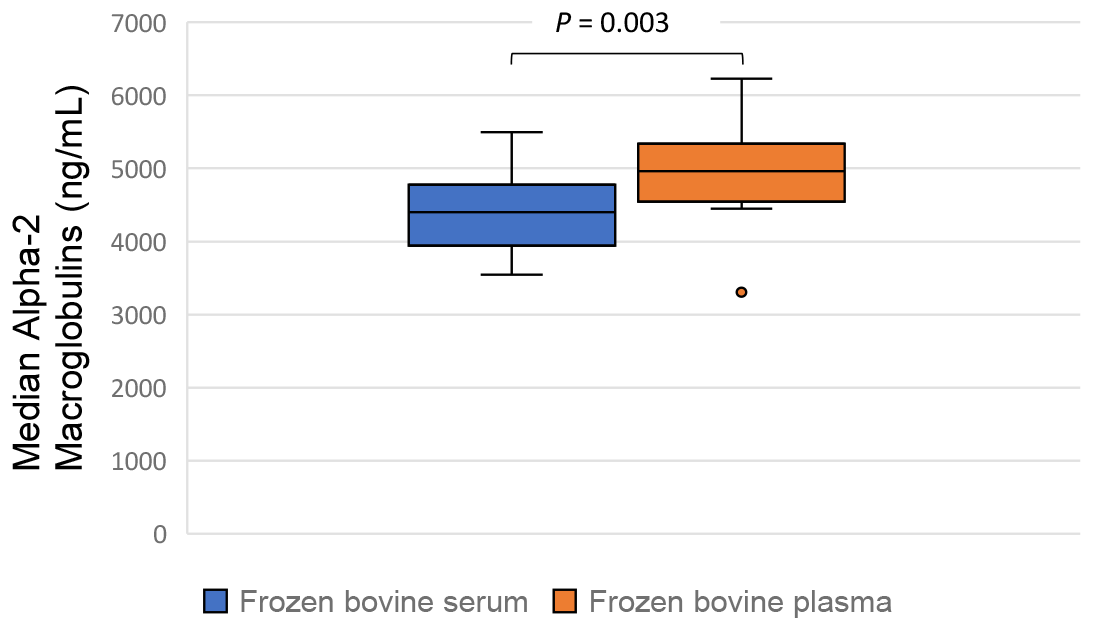
Median alpha-2 macroglobulin concentration (ng/ml) in frozen bovine serum and plasma stored at −20 °C to mimic clinical scenarios. A paired *t*-test was conducted between frozen bovine serum and plasma. A significant mean difference was detected (*P* = 0.003).

### 3.3 Control plates

Bacterial growth was observed in all positive control wells. However, selected wells receiving bovine blood products (without antibiotics) had negative bacterial growth observed (Table 1). For instance, growth of *Staphylococcus pseudintermedius* was inhibited in 98/200 (49%) and 84/200 (42%) of the wells that received bovine frozen plasma and bovine fresh plasma, respectively.

**Table 1:** Number (percent) of wells without bacterial growth, following exposure of bacterial isolates (*n* =10 Staphylococcus pseudintermedius, *n* =10 Streptococcus canis, *n* =10 Pseudomonas aeruginosa) to blood products obtained from 10 healthy bovine.

	Bovine			
Bacteria	Fresh serum	Fresh plasma	Frozen serum	Frozen plasma
*Staphylococcus pseudintermedius*	82/200 (41%)	84/200 (42%)	50/200 (25%)	98/200 (49%)
*Streptococcus canis*	0/200 (0%)	0/200 (0%)	0/200 (0%)	0/200 (0%)
*Pseudomonas aeruginosa*	63/200 (32%)	34/200 (17%)	14/200 (7%)	28/200 (14%)

The bovine blood products were serum or plasma that was fresh or frozen.

### 3.4 Antimicrobial susceptibility testing

Table 2 depicts a representative example of one *Staphylococcus pseudintermedius* isolate, showing the differences in MICs when compared to control and associated changes in clinical interpretation (*i.e,. sensitive* to *intermediate, intermediate* to *resistant, sensitive* to *resistant*). For instance, the clinical interpretation for ceftiofur, chloramphenicol, ciprofloxacin, erythromycin, gentamicin, moxifloxacin and ofloxacin had a decrease in MIC with the addition of each bovine blood product (bS-Fsh, bP-Fsh, bS-Fzn, bP-Fzn) and remained sensitive. Table 3 depicts a representative example of one *Streptococcus canis* isolate, showing the differences in MICs compared to the control and associated changes in clinical interpretation. For example, the clinical interpretation for amikacin, bacitracin, neomycin, and oxytetracycline shifted following an increase in MIC with the addition of each bovine blood product (bS-Fsh, bP-Fsh, bS-Fzn, bP-Fzn). This resulted in a shift from susceptible to non-interpretable for bacitracin, and oxytetracycline. The change in MIC for amikacin resulted in a clinical shift from susceptible to resistant; specifically, the fresh plasma and serum resulted in a clinical shift from susceptible to resistant for neomycin. Tables 4A–C depicts the frequency of *Staphylococcus pseudintermedius* (Table 4A), *Streptococcus canis* (Table 4B), and *Pseudomonas aeruginosa* (Table 4C) isolates exhibiting changes in clinical interpretation (susceptible to resistant or susceptible to intermediate) upon supplementation with each bovine blood product (bS-Fsh, bP-Fsh, bS-Fzn, bP-Fzn). For example, *Pseudomonas aeruginosa* (Table 4C) demonstrated the highest percentages of change in MIC and subsequent change in clinical interpretation (*sensitive* to *resistant*: cefazolin, ceftiofur, ceftiofur, gentamicin and polymyxin B; *sensitive* to *intermediate*: some isolates for ceftiofur, chloramphenicol, and gentamicin). In contrast, *Staphylococcus pseudintermedius* (Table 4A) showed MIC alteration with a lower number of isolates showing a change in clinical interpretation (*sensitive to resistant*: trimethoprim sulfa, a few isolates for ofloxacin, moxifloxacin, gentamicin, erythromycin, ciprofloxacin and ceftiofur; *sensitive to intermediate*: a few isolates for ceftiofur, chloramphenicol, ciprofloxacin, gentamicin and moxifloxacin. Furthermore, *Streptococcus canis* (Table 4B) exhibited only minimal changes in MIC, which resulted in the following clinical interpretations (*sensitive to resistant*: amikacin and neomycin; *sensitive to intermediate*: erythromycin). The general trend for the percentage MIC changes from *sensitive* to *resistant* when plated with the blood products for all bacterial isolates ranged from 4.1% up to 22.9%, whereas the percentage MIC changes from *sensitive* to *intermediate* when plated with blood products ranged from 2.4% up to 9.5%. These trends demonstrate that most blood products did not have any deleterious effects on the MIC when combined with bacteria on the JOEYE2 plates. For each bacterial species (*n* = 10 isolates), results were summarized in Table 5 (*Staphylococcus pseudintermedius*), Table 6 (*Streptococcus canis*), and Table 7 (*Pseudomonas aeruginosa*). Median MICs generally showed no change with the addition of the bovine blood product. However, for certain bacterial isolates, the change in the median MIC was more frequently observed as a decrease (by up to 0.5-fold) with the addition of the blood product to the growth media, though occasionally the MIC increased (up to 4-fold). When data were grouped for all blood products and all bacterial isolates, the overall median (range) fold-change in MICs was 0.5 (0.5–4) (Figure 4).

**Table 2:** Changes in minimal inhibitory concentrations (MICs, in μg/ml) and associated clinical interpretation when one representative bacterial isolate of *Staphylococcus pseudintermedius* was exposed to 17 different ophthalmic antibiotics and either saline (control) or four different blood products originating from healthy bovines.

Antibiotic	Bovine fresh plasma	Bovine fresh serum	Bovine frozen plasma	Bovine frozen serum
Amikacin	16 → 16 NI → NI	16 → 16 NI → NI	16 → 16 NI → NI	16 → 16 NI → NI
Bacitracin	4 → 4 NI → NI	4 → 4 NI → NI	4 → 4 NI → NI	4 → 4 NI → NI
Cefazolin	8 → 8 NI → NI	8 → 8 NI → NI	8 → 8 NI → NI	8 → 8 NI → NI
Ceftiofur	4 → 2 (0.5) S → S	4 → 2 (0.5) S → S	4 → 2 (0.5) S → S	4 → 2 (0.5) S → S
Chloramphenicol	16 → 8 (0.5) S → S	16 → 8 (0.5) S → S	16 → 8 (0.5) S → S	16 → 8 (0.5) S → S
Ciprofloxacin	2 → 1 (0.5) S → S	2 → 1 (0.5) S → S	2 → 1 (0.5) S → S	2 → 1 (0.5) S → S
Doxycycline	0.25 → 0.12 (0.5) NI → NI	0.25 → 0.25 NI → NI	0.25 → 0.12 (0.5) NI → NI	0.25 → 0.25 NI → NI
Erythromycin	1 → 0.5 (0.5) S → S	1 → 0.5 (0.5) S → S	1 → 0.5 (0.5) S → S	1 → 0.5 (0.5) S → S
Gentamicin	4 → 2 (0.5) S → S	4 → 2 (0.5) S → S	4 → 2 (0.5) S → S	4 → 2 (0.5) S → S
Moxifloxacin	1 → 0.5 (0.5) S → S	1 → 0.5 (0.5) S → S	1 → 0.5 (0.5) S → S	1 → 0.5 (0.5) S → S
Neomycin	4 → 4 NI → NI	4 → 4 NI → NI	4 → 4 NI → NI	4 → 4 NI → NI
Ofloxacin	1 → 0.5 (0.5) S → S	1 → 0.5 (0.5) S → S	1 → 0.5 (0.5) S → S	1 → 0.5 (0.5) S → S
Oxytetracycline	1 → 0.5 (0.5) S → NI	1 → 1 NI → NI	1 → 1 NI → NI	1 → 1 NI → NI
Polymyxin B	10 → 10 NI → NI	10 → 10 NI → NI	10 → 10 NI → NI	10 → 10 NI → NI
Ticarcillin	16 → 16 NI → NI	16 → 16 NI → NI	16 → 16 NI → NI	16 → 16 NI → NI
Tobramycin	4 → 4 NI → NI	4 → 4 NI → NI	4 → 4 NI → NI	4 → 4 NI → NI
Trimethoprim	2 → 2 S → S	2 → 2 S → S	2 → 2 S → S	2 → 2 S → S

Boxes highlighted in blue represent a decrease in MIC when compared to control, respectively. S, sensitive; I, intermediate; R, resistant; NI, not interpretable.

**Table 3:** Changes in minimal inhibitory concentrations (MICs, in μg/ml) and associated clinical interpretation when one representative bacterial isolate of *Streptococcus canis* was exposed to 17 different ophthalmic antibiotics and either saline (control) or four different blood products originating from healthy bovine.

Antibiotic	Bovine fresh plasma	Bovine fresh serum	Bovine frozen plasma	Bovine frozen serum
Amikacin	16 → 32 (2) S → R	16 → 32 (2) S → R	16 → 32 (2) S → R	16 → 32 (2) S → R
Bacitracin	2 → 4 (2) S → NI	2 → 4 (2) S → NI	2 → 4 (2) S → NI	2 → 4 (2) S → NI
Cefazolin	8 → 8 NI → NI	8 → 8 NI → NI	8 → 8 NI → NI	8 → 8 NI → NI
Ceftiofur	2 → 2 S → S	2 → 2 S → S	2 → 2 S → S	2 → 2 S → S
Chloramphenicol	4 → 4 NI → NI	4 → 4 NI → NI	4 → 4 S → S	4 → 4 S → S
Ciprofloxacin	1 → 1 NI → NI	1 → 1 NI → NI	1 → 1 NI → NI	1 → 1 NI → NI
Doxycycline	0.5 → 1 (2) NI → NI	1 → 1 NI → NI	1 → 1 NI → NI	1 → 1 NI → NI
Erythromycin	0.5 → 0.5 I → I	0.5 → 0.5 I → I	0.5 → 0.5 I → I	0.5 → 0.5 I → I
Gentamicin	2 → 2 NI → NI	2 → 2 NI → NI	2 → 2 NI → NI	2 → 2 NI → NI
Moxifloxacin	0.5 → 0.5 NI → NI	0.5 → 0.5 NI → NI	0.5 → 0.5 NI → NI	0.5 → 0.5 NI → NI
Neomycin	4 → 8 (2) S → R	4 → 8 (2) S → R	4 → 8 (2) S → NI	4 → 8 (2) S → NI
Ofloxacin	1 → 1 S → S	1 → 1 S → S	1 → 1 S → S	1 → 1 S → S
Oxytetracycline	1 → 4 (4) S → NI	1 → 4 (4) S → NI	1 → 4 (4) S → NI	1 → 4 (4) S → NI
Polymyxin B	10 → 10 NI → NI	10 → 10 NI → NI	10 → 10 NI → NI	10 → 10 NI → NI
Ticarcillin	16 → 16 NI → NI	16 → 16 NI → NI	16 → 16 NI → NI	16 → 16 NI → NI
Tobramycin	4 → 4 NI → NI	4 → 8 (2) NI → NI	4 → 4 NI → NI	4 → 4 NI → NI
Trimethoprim	2 → 2 S → S	2 → 2 S → S	2 → 2 S → S	2 → 2 S → S

Boxes highlighted in red represent an increase in MIC when compared to control, respectively. S, sensitive; I, intermediate; R, resistant; NI, not interpretable.

**Table 4A:** Frequency of *Staphylococcus pseudintermedius* bacterial isolates with changes in clinical interpretation (susceptible to resistance or susceptible to intermediate) with supplementation of bovine fresh plasma, fresh serum, frozen plasma or frozen serum.

STAPHYLOCOCCUS PSEUDINTERMEDIUS				
Antibiotic	Fresh plasma	Fresh serum	Frozen plasma	Frozen serum
Amikacin	0/10	0/10	0/10	0/10
Bacitracin	0/10	0/10	0/10	0/10
Cefazolin	0/10	0/10	0/10	0/10
Ceftiofur	**3/10** ^*^	**3/10** ^*^	3/10^*^, 1/10^*^	1/10^*^, 4/10^*^
Chloramphenicol	**1/10** ^*^	**2/10** ^*^	**1/10** ^*^	**7/10** ^*^
Ciprofloxacin	3/10^*^, 1/10^*^	3/10^*^, 1/10^*^	**3/10** ^*^	3/10^*^, 1/10^*^
Doxycycline	0/10	0/10	0/10	0/10
Erythromycin	**4/10** ^*^	**4/10** ^*^	**4/10** ^*^	**4/10** ^*^
Gentamicin	4/10^*^, 2/10^*^	5/10^*^, 1/10^*^	4/10^*^, 2/10^*^	4/10^*^, 2/10^*^
Moxifloxacin	**3/10** ^*^	2/10^*^, 1/10^*^	**3/10** ^*^	**3/10** ^*^
Neomycin	0/10	0/10	0/10	0/10
Ofloxacin	**3/10** ^*^	**4/10** ^*^	**3/10** ^*^	**3/10** ^*^
Oxytetracycline	0/10	0/10	0/10	0/10
Polymyxin B	0/10	0/10	0/10	0/10
Ticarcillin	0/10	0/10	0/10	0/10
Tobramycin	0/10	0/10	0/10	0/10
Trimethoprim/Sulfa	**8/10** ^*^	**9/10** ^*^	**9/10** ^*^	**9/10** ^*^
#MIC isolate change/total # of isolates (%)	**28****/170** **(16.5%)**; **4****/170** **(2.4%)**	**30****/170** **(17.6%)**; **5****/170****(2.9%)**	**27****/170****(15.9%)**; **6****/170****(3.5%)**	**27****/170****(15.9%)**; **14****/170****(8.2%)**
^*^MIC change to Resistant, ^*^MIC change to Intermediate, **no change in MIC**.				

Bolded red with asterisk = MIC change to resistant; Bolded orange with asterisk = MIC change to intermediate; Black = no change in MIC. The number of MIC isolate change/total number of isolates (%) is shown in the bottom row of the table.

**Table 4B:** Frequency of *Streptococcus canis* bacterial isolates with changes in clinical interpretation (susceptible to resistance or susceptible to intermediate) with supplementation of bovine fresh plasma, fresh serum, frozen plasma or frozen serum.

STREPTOCOCCUS CANIS				
Antibiotic	Fresh Plasma	Fresh Serum	Frozen Plasma	Frozen Serum
Amikacin	**7/10** ^*^	**7/10** ^*^	**7/10** ^*^	**7/10** ^*^
Bacitracin	0/10	0/10	0/10	0/10
Cefazolin	0/10	0/10	0/10	0/10
Ceftiofur	0/10	0/10	0/10	0/10
Chloramphenicol	0/10	0/10	**2/10** ^*^	**1/10** ^*^
Ciprofloxacin	0/10	0/10	0/10	0/10
Doxycycline	0/10	0/10	0/10	0/10
Erythromycin	**10/10** ^*^	**10/10** ^*^	**10/10** ^*^	**10/10** ^*^
Gentamicin	0/10	0/10	0/10	0/10
Moxifloxacin	0/10	0/10	0/10	0/10
Neomycin	**10/10** ^*^	**10/10** ^*^	0/10	0/10
Ofloxacin	0/10	0/10	0/10	0/10
Oxytetracycline	0/10	0/10	0/10	0/10
Polymyxin B	0/10	0/10	0/10	0/10
Ticarcillin	0/10	0/10	0/10	0/10
Tobramycin	0/10	0/10	0/10	0/10
Trimethoprim/Sulfa	0/10	0/10	0/10	0/10
#MIC isolate change/total # of isolates (%)	**17****/170****(10%)**; **10****/170** **(5.9%)**	**17****/170****(10%)**; **10****/170****(5.9%)**	**7****/170****(4.1%)**; **12****/170****(7.1%)**	**7****/170****(4.1%)**; **11****/170****(6.5%)**
^*^MIC change to Resistant, ^*^MIC change to Intermediate, **no change in MIC**				

Bolded red with asterisk = MIC change to resistant; Bolded orange with asterisk = MIC change to intermediate; Black = no change in MIC. The number of MIC isolate change/total number of isolates (%) is shown in the bottom row of the table.

**Table 4C:** Frequency of *Pseudomonas aeruginosa* bacterial isolates with changes in clinical interpretation (susceptible to resistance or susceptible to intermediate) with supplementation of bovine fresh plasma, fresh serum, frozen plasma or frozen serum.

PSEUDOMONAS AERUGINOSA				
Antibiotic	Fresh plasma	Fresh serum	Frozen plasma	Frozen serum
Amikacin	**0/10**	**1/10** ^*^	**0/10**	**0/10**
Bacitracin	**0/10**	**0/10**	**0/10**	**0/10**
Cefazolin	**9/10** ^*^	**10/10** ^*^	**10/10** ^*^	**10/10** ^*^
Ceftiofur	9/10^*^, 1/10^*^	**9/10** ^*^	**10/10** ^*^	**10/10** ^*^
Chloramphenicol	1/10^*^, 7/10^*^	1/10^*^, 4/10^*^	2/10^*^, 6/10^*^	2/10^*^, 6/10^*^
Ciprofloxacin	**0/10**	**0/10**	**0/10**	**0/10**
Doxycycline	**0/10**	**0/10**	**0/10**	**0/10**
Erythromycin	**0/10**	**0/10**	**0/10**	**0/10**
Gentamicin	**1/10** ^*^ **, 8/10** ^*^	**6/10** ^*^	**7/10** ^*^ **, 3/10** ^*^	**4/10** ^*^ **, 6/10** ^*^
Moxifloxacin	**0/10**	**0/10**	**0/10**	**0/10**
Neomycin	**0/10**	**0/10**	**0/10**	**0/10**
Ofloxacin	**0/10**	**0/10**	**0/10**	**0/10**
Oxytetracycline	**0/10**	**0/10**	**0/10**	**0/10**
Polymyxin B	**9/10** ^*^	**10/10** ^*^	**10/10** ^*^	**10/10** ^*^
Ticarcillin	**0/10**	**0/10**	**0/10**	**0/10**
Tobramycin	**0/10**	**0/10**	**0/10**	**0/10**
Trimethoprim/sulfa	**0/10**	**0/10**	**0/10**	**0/10**
#MIC isolate change/total # of isolates (%)	**29****/170****(17.1%)**; **16****/170****(9.5%)**	**31****/170****(18.2%)**; **10****/170** **(5.9%)**	**39****/170****(22.9%)**; **9****/170****(5.3%)**	**36****/170****(21.2%)**; **12****/170****(7.1%)**
^*^MIC change to Resistant, ^*^MIC change to Intermediate, **no change in MIC**.				

Bolded red with asterisk = MIC change to resistant; Bolded orange with asterisk = MIC change to intermediate; Black = no change in MIC. The number of MIC isolate change/total number of isolates (%) is shown in the bottom row of the table.

**Table 5:** Median changes in minimal inhibitory concentrations (MICs) of ophthalmic antibiotics used all 10 against *Staphylococcus pseudintermedius* isolates from canine patients with infectious keratitis.

Antibiotic	Bovine fresh plasma	Bovine fresh serum	Bovine frozen plasma	Bovine frozen serum	*P*–value
Amikacin	–	–	–	–	–
Bacitracin	–	–	–	–	–
Cefazolin	–	–	–	–	–
Ceftiofur	0.5	0.5	0.5	0.75	0.194
Chloramphenicol	0.5	0.5	0.5	–	**0.001** ^*^
Ciprofloxacin	0.5	0.5	0.5	0.5	0.3916
Doxycycline	–	–	–	–	0.3916
Erythromycin	0.5	0.5	0.5	0.5	–
Gentamicin	2	2	2	2	–
Moxifloxacin	0.5	0.5	0.5	0.5	–
Neomycin	–	–	–	–	–
Ofloxacin	0.75	–	–	0.75	0.3916
Oxytetracycline	–	–	–	–	**0.044** ^*^
Polymyxin B	–	–	–	–	–
Ticarcillin	–	–	–	–	–
Tobramycin	–	–	–	–	0.1116
Trimethoprim	–	–	–	–	–

Boxes highlighted in red or blue represent an increase or decrease in MIC when compared to control, respectively. *P*-values represent statistical comparisons among all groups (Friedman rank sum test). Bolded text and asterisks indicate statistical differences of a specific blood product vs. control (*post-hoc* Nemenyi test).

Results are depicted as fold change in median MIC when comparing standard test medium (*i.e.*, no blood product = control) *vs*. test medium supplemented with bovine blood products (serum or plasma; fresh or frozen). – indicates no change in MIC.

**Table 6:** Median changes in minimal inhibitory concentrations (MICs) of ophthalmic antibiotics used against all 10 *Streptococcus canis* isolates from canine patients with infectious keratitis.

Antibiotic	Bovine fresh plasma	Bovine fresh serum	Bovine frozen plasma	Bovine frozen serum	*P*-value
Amikacin	2	2	2	2	–
Bacitracin	–	–	–	–	–
Cefazolin	–	–	–	–	–
Ceftiofur	0.5	0.5	0.5	0.5	–
Chloramphenicol	–	–	–	–	–
Ciprofloxacin	–	–	–	–	–
Doxycycline	1.5	2.5	4	4	0.065
Erythromycin	–	–	–	–	–
Gentamicin	–	–	–	–	–
Moxifloxacin	–	–	–	–	–
Neomycin	–	–	–	–	–
Ofloxacin	0.5	0.5	0.5	0.5	–
Oxytetracycline	–	–	–	–	–
Polymyxin B	–	–	–	–	–
Ticarcillin	–	–	–	–	–
Tobramycin	0.67	1.33	0.67	1.33	**0.001** ^*^
Trimethoprim	–	–	–	–	–

Bolded text and asterisks indicate statistical differences of a specific blood product vs. control (*post-hoc* Nemenyi test).

Results are depicted as fold change in median MIC when comparing standard test medium (*i.e.*, no blood product = control) *vs*. test medium supplemented with bovine blood products (serum or plasma; fresh or frozen). – indicates no change in MIC. Boxes highlighted in red or blue represent an increase or decrease in MIC when compared to control, respectively. *P*-values represent statistical comparisons among all groups (Friedman rank sum test).

**Table 7:** Median changes in minimal inhibitory concentrations (MICs) of ophthalmic antibiotics used against all 10 *Pseudomonas aeruginosa* isolates from canine patients with infectious keratitis.

Antibiotic	Bovine fresh plasma	Bovine fresh serum	Bovine frozen plasma	Bovine frozen serum	*P*–value
Amikacin	–	–	–	–	0.3916
Bacitracin	–	–	–	–	–
Cefazolin	2	2	2	2	–
Ceftiofur	2	2	2	2	0.3916
Chloramphenicol	–	–	–	–	0.2699
Ciprofloxacin	–	–	–	–	–
Doxycycline	–	–	–	–	0.3916
Erythromycin	–	–	–	–	–
Gentamicin	–	–	2	–	**0.0002** ^*^
Moxifloxacin	–	–	–	–	–
Neomycin	–	–	–	–	0.194
Ofloxacin	0.5	0.5	0.5	0.5	0.6149
Oxytetracycline	–	–	–	–	0.2839
Polymyxin B	–	–	–	–	–
Ticarcillin	–	–	–	–	–
Tobramycin	–	–	–	–	–
Trimethoprim	–	–	–	–	–

Bolded text and asterisks indicate statistical differences of a specific blood product vs. control (*post-hoc* Nemenyi test).

Results are depicted as fold change in median when comparing standard test medium (*i.e.*, no blood product = control) *vs*. test medium supplemented with bovine blood products (serum or plasma; fresh or frozen). – indicates no change in MIC. Boxes highlighted in red or blue represent an increase or decrease in MIC when compared to control, respectively. *P*-values represent statistical comparisons among all groups (Friedman rank sum test).

**Figure 4:**
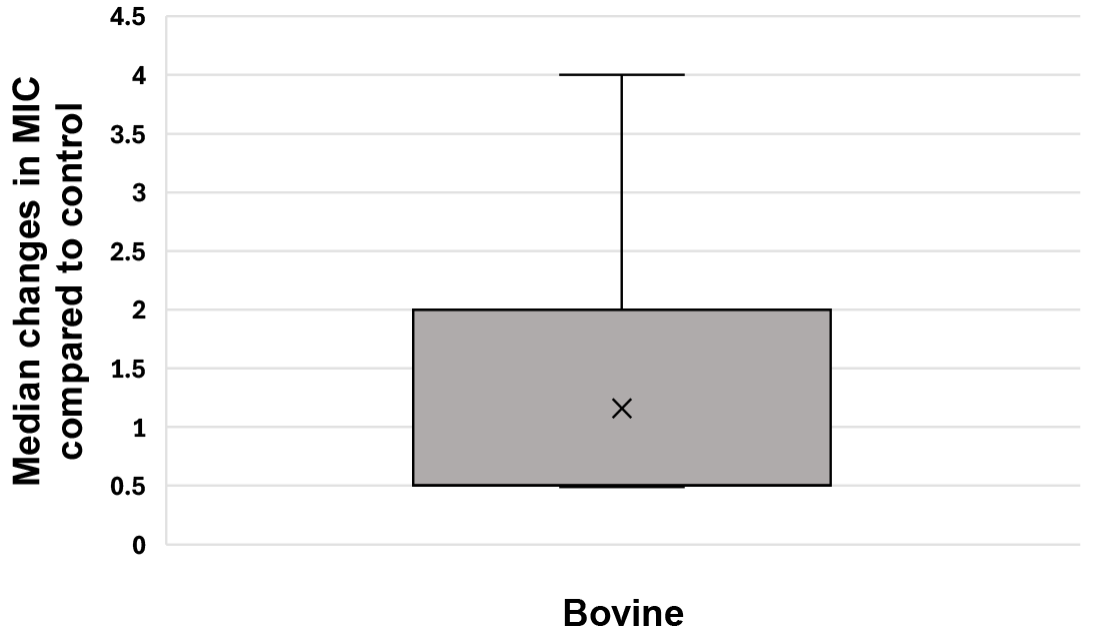
Box-and-whisker plots depict changes in minimal inhibitory concentrations (MICs) when comparing control (saline) to bovine blood products (plasma and serum; fresh and frozen) for all bacterial isolates combined. Median values are shown by an X. First and third quartiles (25th and 75th percentiles) are represented by the lower and upper limits of the box, respectively. The 2.5th and the 97.5th percentiles are shown as the lower and upper whiskers, respectively.

## 4 Discussion

Bovine blood products modulated the MICs of ophthalmic antibiotics against common ocular pathogens previously isolated from canines with bacterial keratitis. This effect varied with the specific bacterial species, type of blood product (serum or plasma), storage condition (fresh or frozen), and antibiotic; with some MICs increased in the presence of bovine blood products (up to 4-fold), while others decreased (down to 0.5-fold) for selected antibiotics.

The elevations in MICs noted in this study were lower than those reported for canine and equine blood products (up to 10.8-fold and up to 5-fold increase in MIC, respectively), (11) but were likely impacted by the presence of albumin in bovine serum and plasma. This is since albumin concentration was much higher than the minimum albumin level (0.5 mg/mL) previously shown to impact the efficacy of selected ophthalmic antibiotics against common ocular pathogens (10). Albumin levels in canine and equine blood products (11) were higher than those quantified on the ocular surface of dogs with spontaneously occurring breakdown of the blood-tear barrier (10, 15); however, the bovine albumin concentration, was within the range of albumin concentration in dogs with naturally occurring blood-tear barrier breakdown. This is clinically relevant since topical antibiotics will mix with a topical blood product (containing albumin) on the ocular surface, causing the bound portion of the antibiotic to become metabolically inactive (8, 9), thereby increasing the amount of antibiotic required to inhibit bacterial growth. Clinically, the increased MICs associated with blood products may contribute to poorer treatment outcomes, as a recent study reported deterioration of bacterial keratitis in canine patients despite aggressive antibiotic therapy (10). Previous human studies have also shown increased scarring and delayed wound healing with higher MICs (16, 17). There is concern that increased MICs from blood products may result in a greater antibiotic resistance, due to bacterial exposure to sub-therapeutic antibiotic concentrations, resulting in increased potential antibiotic resistance (18), which threatens both veterinary and human ophthalmology patients (12, 19, 20). An increase in MIC was observed in a small percentage of bacterial isolates upon the addition of bovine blood products, altering the clinical interpretation from sensitive to intermediate or resistant. Despite the low frequency of this occurrence, it remains clinically significant due to concerns regarding increased antimicrobial resistance in these clinical scenarios. In clinical practice, the MIC is used to determine the susceptibility of a microorganism to an antimicrobial agent, whereas the minimum bactericidal concentration (MBC) is the lowest antimicrobial concentration that completely kills the microorganism. The MBC/MIC ratio is key for determining whether an antimicrobial agent *in vitro* is bactericidal (ratio ≤ 4) or bacteriostatic (>4), which is essential for the complete resolution of a severe microbial infection (21).

Reductions in MICs were observed more regularly with bovine blood products; it is hypothesized that these reductions may be related to natural antimicrobial peptides (e.g., lysozymes) within the serum or plasma (22). Antimicrobial effects were observed with bovine serum and plasma (fresh or frozen) when plated with *Staphylococcus pseudintermedius* and to a lesser extent with *Pseudomonas aeruginosa*. This observation was similar to that of a previous study where equine serum (to a lesser extent) and equine plasma had a greater antimicrobial effect compared to canine blood products when plated with *Staphylococcus pseudintermedius* and *Pseudomonas aeruginosa* (11). Another study by Yates et al. assessed the antimicrobial efficacy of equine and canine autologous conditioned plasma and serum along with amniotic membrane drops against several ocular pathogens including *Streptococcus spp*., *Staphylococcus spp*., *Pseudomonas aeruginosa* and other bacterial species (*Enterococcus faecalis, E. coli, Bacillus cereus*, etc.) (23). The results of that study showed that the antimicrobial effect of autologous conditioned plasma only partially inhibited only *E. faecalis*, while no antimicrobial effects were observed from autologous conditioned serum, plasma, or amniotic membrane drops against any of the other bacterial isolates. These two studies, along with our results, demonstrate that antimicrobial effects may depend on the type of blood product (plasma or serum), species of origin, and the type of bacterial pathogen. Antimicrobial properties present in blood products may help to explain the lower MICs observed in this bovine blood product study and those with previously evaluated equine and canine blood products (12). There is still much work to be done to further elucidate the specific mechanisms underlying the antimicrobial effects within blood products, and further proteomic investigation will hopefully help to discern the responsible factors in serum and plasma.

Bacterial keratitis is a severe, vision threatening disease process in both human and veterinary patients (24). Corneal infections require rapid diagnosis and aggressive medical management to reduce the damaging effects of the infection while promoting wound healing. Blood products are known to be beneficial in the management of infectious keratitis. First, they contain alpha-2 macroglobulins and alpha-1 antitrypsin, which help mitigate collagenolytic activity from MMPs and serine proteinases on the ocular surface (1, 25–28). Second, they contain essential growth factors that can aid in corneal wound healing, such as epidermal growth factor, platelet-derived growth factors, transforming growth factor beta, insulin-like growth factor, vascular endothelial growth factor and fibroblastic growth factor (29). However, it is important to recognize that blood products do not appear to speed up healing of corneal epithelial defects (30, 31). Furthermore, simultaneous use of antibiotics and blood products may result in interference, as demonstrated by the increase in MICs for some ocular pathogens tested in this study and a previous study (11).

However, bovine blood products seem to have a reduced effect on the MIC compared to equine and canine blood products. This may be due to significantly lower albumin concentration detected in bovine fresh/frozen serum and plasma (2 mg/ml) compared to canine (14 mg/ml) and equine (25 mg/ml) (11). The albumin concentration in this study was surprisingly low, which may be explained by malnutrition or inadequate protein intake vs. protein loss through the gastrointestinal system (32). Other causes of hypoalbuminemia include stage of lactation, liver disease, renal disease and other systemic inflammatory (e.g., mastitis, metritis or peritonitis) diseases, though none of the 10 sampled bovine were clinically unhealthy or obviously malnourished. These results may warrant future comparative testing of the bovine albumin ELISA kit against standard bovine blood serum albumin levels (normal range = 2.5–3.8 g/dl). Another potential cause for the lower albumin levels could be storage effects, such as the degradation of albumin during refrigeration or freezing, however this scenario seems less likely.

The main clinical take-home points of this study reflect those of the previous study evaluating equine and canine blood products (11): (1) Use either fresh or frozen blood products as there was no difference in albumin concentration or antibiotic efficacy between the two storage conditions; (2) Apply topical blood products last, at least 15 min or longer, after last topical antibiotics. The rationale for waiting this duration is that it minimizes the negative impact of albumin-antibiotic binding, as less than 5% of the drug will remain in the tear film at that time (33) and (3) Bovine blood products may be preferred over equine and canine products because they have less apparent effect on antimicrobial susceptibility; however, direct comparative studies are warranted in the future.

The main limitation of this study is its' *in vitro* nature, which does not fully reflect the dynamics observed in clinical patients with bacterial keratitis. For example, clinical patients often experience increased tearing or discomfort, resulting in accelerated loss of topical antibiotics through the nasolacrimal system or overflow onto the face (34, 35). Furthermore, sourcing samples from a single population of dairy cattle from a solitary location introduced the possibility of highly abnormal albumin values, which may not fully represent the broader cattle population. Additionally, the scope was restricted by the low number of bacterial samples used, as we utilized only 10 isolates from each bacterial species. Consequently, this small sample size does not represent all the isolates encountered in a clinical setting. An inherent limitation of utilizing biological blood products is the potential presence of background pyrogens or endotoxins. While strict aseptic protocols were followed during sample processing to mitigate this risk, baseline pyrogenic activity remains a variable inherent to *ex-vivo* blood product evaluations that could theoretically influence microbial interactions, specifically potentially skewing the MIC results. Another potential limitation of this study involves the application of standard clinical breakpoints, which are typically established for systemic antimicrobial therapy rather than topical treatment. As noted in CLSI VET09, isolates categorized as intermediate or resistant may still respond to topical administration due to higher local drug concentrations achieved *in vivo*. However, in patients with infectious keratitis, these high concentrations can be significantly depleted through mechanisms such as tear film albumin binding, reflex tearing, and drainage via the nasolacrimal system. Consequently, such losses may reduce effective drug bioavailability, potentially limiting the clinical efficacy of topical therapy. A final limitation is that we only focused on two blood products from bovine to compare with the previous study on equine and canine blood products (11). There are other blood product types (e.g., platelet rich plasma) or blood products from other species that warrant future investigation (36, 37).

In summary, bovine blood products have less of an effect on the MICs of ophthalmic antibiotics against common ocular pathogens isolated from canine patients with bacterial keratitis. When using blood products, clinicians should consider using bovine blood products due to their minimal impact on antimicrobial susceptibility and favorable antimicrobial effects. Ideally, these blood products should be administered at least 15 min after the last topical antibiotic to minimize albumin-antibiotic binding within the tear film, thereby maximizing antibiotic efficacy.

## Data Availability

The raw data supporting the conclusions of this article will be made available by the authors, without undue reservation.
